# Evaluation of formalin-fixed paraffin-embedded tissues in the proteomic analysis of parathyroid glands

**DOI:** 10.1186/1477-5956-9-29

**Published:** 2011-06-08

**Authors:** Elena Donadio, Laura Giusti, Filomena Cetani, Ylenia Da Valle, Federica Ciregia, Gino Giannaccini, Elena Pardi, Federica Saponaro, Liborio Torregrossa, Fulvio Basolo, Claudio Marcocci, Antonio Lucacchini

**Affiliations:** 1 Department of Psychiatry, Neurobiology, Pharmacology and Biotechnology, University of Pisa, Via Bonnanno 6, Pisa, 56126, Italy; 2 Department of Endocrinology and Metabolism, Orthopedics and Traumatology, Occupational Medicine University of Pisa, Via Paradisa 2, Pisa, 56124, Italy; 3 Department of Surgery, University of Pisa, Via Paradisa 2, Pisa, 56124, Italy

## Abstract

**Background:**

Proteomic research in the field of parathyroid tissues is limited by the very small dimension of the glands and by the low incidence of cancer lesions (1%). Formalin-fixed paraffin-embedded (FFPE) tissue specimens are a potentially valuable resource for discovering protein cancer biomarkers. In this study we have verified the applicability of a heat induced protein extraction from FFPE parathyroid adenoma tissues followed by a gel-based or gel-free proteomic approach in order to achieve protein separation and identification.

**Results:**

The best results for high quality MS spectra and parameters, were obtained by using a gel-free approach, and up to 163 unique proteins were identified. Similar results were obtained by applying both SDS-out and SDS-out + TCA/Acetone techniques during the gel-free method. Western blot analysis carried out with specific antibodies suggested that the antigenicity was not always preserved, while specific immunoreactions were detected for calmodulin, B box and SPRY domain-containing protein (BSPRY), peroxiredoxin 6 (PRDX 6) and parvalbumin.

**Conclusions:**

In spite of some limitations mainly due to the extensive formalin-induced covalent cross-linking, our results essentially suggest the applicability of a proteomic approach to FFPE parathyroid specimens. From our point of view, FFPE extracts might be an alternative source, especially in the validation phase of protein biomarkers when a large cohort of samples is required and the low availability of frozen tissues might be constraining.

## Background

Proteome analysis provides diagnostic information that can be essential for therapeutic predictions. Recently, we have performed a proteomic study of parathyroid glands by using a two-dimensional electrophoresis (2-DE)/mass spectrometry (MS)-based approach, and we have examined the global changes of the parathyroid adenoma tissues protein profile compared to normal parathyroid tissues [1]. This application has resulted in the identification of a panel of proteins, which are differentially expressed, and has suggested that the proteomic approach might be useful in identifying potential biomarkers in parathyroid carcinoma. Although the use of fresh materials is desirable for any analytical technology, large cohorts of fresh-frozen human tissue samples are often difficult to acquire or might not be large enough to accurately represent the tumor origin. For example, in our experience, proteomic research studies in the field of parathyroid tumors strictly depend on the availability of parathyroid tissues (*i.e*. very small dimension of the glands, or low incidence of cancer lesions) both for normal and cancer samples, prompting us to find an alternative source of fresh and frozen tissues. Formalin fixed paraffin embedded (FFPE) tissues, a common tissue preservation technique, is routinely performed for the preparation of samples used for pathological analysis. The enormousness of the FFPE tissue blocks represents an invaluable and largely untapped resource for testing biomarkers and discovering new ones. For this reason, besides the use of this resource in immunohistochemistry (IHC) and *in situ *hybridization-based analysis, it would be valuable to complement IHC with powerful high resolution methods and mass spectrometry-based proteomics in order to utilize this archived material as an alternative resource for research purposes. However, proteins in FFPE tissue during the fixation process undergo degradation and cross-linking, and have therefore long been thought to be unsuitable for MS-based proteomics. Only in the past few years, the identification of a few hundred or thousand proteins in FFPE tissues has been reported [2-32]. Due to protein degradation and cross-linking caused by formalin fixation, several studies have been conducted to test the applicability of other fixatives to proteomic analysis [2-26]. In these studies, proteins were extracted from ethanol-fixed [2], Fine-FIX fixed [3] and HOPE-fixed [4] paraffin embedded tissues and resolved by 2-DE. Protein profiles obtained by using these formalin-free fixatives were highly similar to those obtained by fresh-frozen tissues. Moreover, both the duration of formalin fixation [5] and tissue storage [6] have been taken into account to assess the quality of the protein extracts. In order to improve protein extraction, many protocols have been developed to extract full-length proteins [7-11] or tryptic peptides [11-14]. The quality and identity of full-length proteins have been evaluated by SDS-PAGE, Western Blot [4,8-10] and 2-DE [10,11,15], while tryptic peptides have been identified by LC-MS/MS [6,11] or by CIEF/Nano RLPC multidimensional peptide separation [16]. In other studies, extracted proteins have been directly analyzed by MALDI-Imaging [14,17-19], and the results were comparable with those obtained by MALDI-Tof MS and nLC-MS [17]. Moreover, this strategy is suitable for FFPE tissues conserved for a long time [18]. Recently, relative and absolute protein expressions have been successfully estimated for quantitative proteomic analysis [20-26]. This proteomic application appears to be useful for biomarker discovery from FFPE tumor tissues in comparison to healthy ones [24].

In this report, we utilized an easily accessible extraction buffer coupled with a heat-induced antigen retrieval technique to obtain proteins from FFPE tissue specimens of parathyroid adenoma. The separation of proteins extracted from the FFPE tissues was performed by both gel-free and gel-based proteomics. The protein identifications were validated by western blot analysis and compared with those obtained with fresh-frozen tissues.

## Materials and methods

### Materials

Iodoacetamide (IAA), dithiothreitol (DTT), 3-[(3-Cholamidopropyl)dimethylammonio]-1-propanesulfonate (CHAPS), urea, thiourea, glycerol, sodium dodecyl sulfate (SDS), tetramethylethylenediamine (TEMED), ammonium persulfate, glycine and 30% acrylamide-N,N,N bisacrylamide were acquired from Applichem (Germany). IPGs pH 3-10 NL, pharmalyte 3-10, dry strip cover fluid and the enhanced chemiluminescence (ECL) detection system were purchased from GE Health Care Europe (Uppsala, Sweden). The SDS-out precipitation kit was purchased from Thermo Scientific Rockford (IL, USA). The anti-Parvalbumin, anti-PRDX-6, anti-calmodulin, anti-MAPK1, and anti-beta actin specific primary antibodies and secondary antibodies (horseradish peroxidase (HRP)-conjugated) were from Santa Cruz Biotecnology (CA, USA). Anti-CCT-5, anti-PARK7 and anti-BSPRY specific primary antibodies were from the Abnova Corporation (Taiwan). All other reagents were from standard commercial sources and were of the highest grade available.

### Protein extraction

Specimens of FFPE adenoma parathyroid tissues of five patients with sporadic primary hyperparathyroidism (PHPT) were collected from the tissue bank of the pathology section of the Department of Surgery of the University of Pisa. The study was approved by the local Ethics Committee. All patients gave their informed consent for the study. Seven to fifteen 5 μm section pieces obtained from each patient were pooled and deparaffinized in 2-5 changes of xylene for 10 min each. Then the tissues were rehydrated through a series of graded ethanol (100% two times, 85%, 70%) for 10 min each. After rehydration, the tissues were resuspended in an extraction buffer of 20 mM Tris-HCl at pH 4 containing 2% SDS and 0.2 M glycine, sonicated 3 times for 10 sec each and incubated at 4°C for 1 hour under agitation. The homogenates were heated at 100°C for 20 min and successively at 60°C for 2 hours. The crude extracts were finally clarified by centrifugation at 13000 *g *for 20 min at 4°C. The same procedure was performed for different section pieces, only changing the pH values of the extraction buffer (pH 6 and 9). Three different extractions of pooled samples were performed at each pH value. The protein content of the extracts was determined by RC/DC of Biorad using albumin as the standard. For the gel-free approach, the proteins from the FFPE specimens were extracted in buffer at pH 6 as above described. Then the protein extracts were treated to reduce the content of SDS as described below to obtain the SDS-free samples. An aliquot of these samples were lyophilized in a speed-vac (SDS-Out samples). Alternatively, an aliquot of SDS-free samples was precipitated using 10% (w/v) TCA and 0.05% DTT. After incubation at 0°C for one hour, insoluble material was pelleted at 14000 g. Pellets were washed three times with cold pure acetone and air dried (SDS-Out + TCA/Acetone samples). The experiments were performed in triplicate.

### SDS-Out precipitation kit

In order to remove the excess SDS from the samples, the SDS-Out precipitation kit was used following the manufacture's instructions. Briefly, 1 volume of SDS-Out precipitation reagent was added to 20 volumes of sample. Then the mixture was incubated in ice for 20 min and centrifuged at 10000 *g *for 10 minutes at room temperature. The supernatant was transferred to a spin cup column and centrifuged for 1 minute at 10000 *g *to clarify the supernatant. The samples were stored at -80°C until they were used.

### 2-DE

2-DE was performed using the Immobiline-polyacrylamide system as previously described [1]. For analytical gels, 150 μg of protein from each preparation was filled to 350 μl with a rehydration buffer supplemented with 1% (v/v) pharmalytes, and pH range from 3-10. The first dimension was run on a non-linear wide range immobilized pH gradient IPG (pH range from 3-10, 18 cm), at 16°C on an Ettan IPGphor II apparatus (GE Health Care) according to the procedure previously described [27]. After equilibration, the strips were applied to the top 12% SDS-PAGE gels, and electrophoresis was performed using the PROTEAN-II Multi Cell system (Bio-Rad) with constant amperage (40 mA/gel) at 10°C until the front dye reached the bottom of the gel. For the preparative run, 300 μg of protein from the extract, free from SDS, was loaded. Analytical gels were stained by using ammoniacal silver nitrate as previously described [28]. Alternatively, a silver stain protocol compatible with mass spectrometry analysis was performed on the preparative gels [29]. The stained gels were scanned with an Epson Expression 1680 Pro scanner, and the images were analyzed with the Image-Master 2D Platinum 6.01 (GE Health Care) software program. For the analysis, the samples were grouped into classes depending on the pH values. Protein spots were detected and matched among different samples. Individual spot volume values were obtained according to the program instructions. The normalized protein abundance was calculated as the spot intensity for an individual spot divided by the spot intensity for all valid spots, and then reported as a percentage of the volume.

### LC-ESI-MS/MS analysis

#### Gel samples treatment for mass spectrometry

Fragments of the gel containing proteins of interest were washed for 30 min with 100 μl of 50 mM ammonium bicarbonate and 30% AcN. Gel pieces were then dried for 30 minutes in a Hetovac vacuum centrifuge (HETO, Allerod, Denmark). Dried pieces of gel were rehydrated for 45 min at 4°C in 5-20 μl of a 50 mM ammonium bicarbonate solution containing sequencing grade trypsin at 6.25 ng/μl (Promega, Madison, WI, USA). After overnight incubation at 37°C, gel pieces were dried in a high vacuum centrifuge before being rehydrated by the addition of 20 μl of H_2_O, and finally dried again. Elution of the peptides was performed with 20 μl of 1% TFA for 20 min at room temperature with occasional shaking. The TFA solution containing the proteins was transferred to a polypropylene tube. A second elution of the peptides was performed with 20 μl of 0.1% TFA in 50% AcN for 20 min at room temperature with occasional shaking. The second TFA solution was pooled with the first one. The volume of the pooled extracts was reduced to 1-2 μl by evaporation under vacuum. Control extractions (blanks) were performed using pieces of gels devoid of proteins.

#### Lyophilized samples treatment for mass spectrometry

Lyophilized samples were suspended in 100 ml of distilled water., Thirty μl of urea (6 M in 50 mM ammonium bicarbonate) were added to 30 μl of this solution, and the mixture was incubated at 37°C for 30 min. Then, 30 μl of DTT (38 mM in distilled water) were added to the mixture, and the reduction was carried out at 37°C for 1 h. Alkylation was performed by adding 60 μl of iodoacetamide (108 mM in 50 mM ammonium bicarbonate) during 1 hour at room temperature in the dark. Five μl of trypsin porcine (Sigma) solution (50 ng/μl in 50 mM ammonium bicarbonate) was added, and the digestion was proceeded overnight at 37°C. The sample was desalted with a C18 microspin column (Harvard Apparatus, Holliston, MA, USA), dried, and re-dissolved in H_2_0/CH_3_CN/FA 94.9/5/0.1 before LC-ESI-MS/MS analysis.

LC-ESI-MS/MS was performed on a linear trap quadrupole (LTQ) Orbitrap XL from Thermo Electron (San Jose, CA, USA) equipped with a NanoAcquity system from Waters. Peptides were trapped on a home-made 5 μm 200 Å Magic C18 AQ (Michrom) 0.1 × 20 mm pre-column and separated on a home-made 5 μm 100 Å Magic C18 AQ (Michrom) 0.75 × 150 mm column with a gravity-pulled emitter. The analytical separation was run for 35 min using a gradient of H_2_O/FA 99.9%/0.1% (solvent A) and CH_3_CN/FA 99.9%/0.1% (solvent B). The gradient was run as follows: 0-13 min 95% A and 5% B, then to 65% A and 35% B at 14 min, and 20% A and 80% B at 19 min at a flow rate of 220 nL/min. For the MS survey scans, the orbitrap (OT) resolution was set to 60000, and the ion population was set to 5 × 10^5 ^with an m/z window from 400 to 2000. For protein identification, up to five precursor ions were selected for collision-induced dissociation (CID). For MS/MS in the LTQ, the ion population was set to 1 × 10e4 (isolation width of 2 m/z), while for MS/MS detection in the OT, it was set to 2 × 10e5 (isolation width of 4 m/z) and 1 × 10e5 (isolation width of 2 m/z) for gel samples and lyophilized samples, respectively, with resolution of 7500, first mass at m/z = 100, and maximum injection time of 750 ms. The normalized collision energies were set to 35% for CID.

### Database searching and criteria for protein identification

Peak lists were generated from raw orbitrap data using the embedded software from the instrument vendor (extract_MSN.exe). The monoisotopic masses of the selected precursor ions were corrected using an in-house written Perl script [30]. The peak list files were searched against the SwissProt/trEMBL database (Release 15.10 of 03-Nov-2009) using Mascot (Matrix Sciences, London, UK). Human taxonomy (98529 sequences) was specified for database searching. The parent ion tolerance was set to 10 ppm. Variable amino acid modifications were oxidized methionine. Trypsin was selected as the enzyme, with one potential missed cleavage, and the normal cleavage mode was used. The mascot search was validated using Scaffold 3.00.03 (Proteome Software, Portland, OR). Only proteins matching with two different peptides with a minimum probability score of 95% were considered identified.

### Western blot

Western blot analysis was essentially performed as previously described [1]. Fresh/frozen tissues of PHPT patients (n = 3) were obtained at surgery, immediately snap frozen in liquid nitrogen, and stored at -80°C until use. Both? Aliquots of proteins (25 μg) from FFPE pooled tissues extracted at pH 6 and from fresh/frozen pooled adenoma tissues were subjected to SDS-PAGE and transferred to nitrocellulose, blocked with PBS/tween/milk and then incubated with specific polyclonal antibodies: anti-calmodulin (dilution 1:200), anti-PARK7 (dilution 1:200), anti-MAPK-1 (dilution 1:200), and anti-parvalbumin α (dilution 1:200); anti-CCT-5 (5 μg/ml); anti-β actin (dilution 1:1000), anti-BSPRY (dilution 1:500) and anti-PRDX6 (dilution 1:200). Antibody reactivity was detected using HRP-conjugated anti-rabbit or anti-mouse antibodies and visualized with a chemiluminescent peroxidase substrate (ECL system).

## Results

### Comparing FFPE parathyroid adenoma tissues extractions at different pH values using 2-DE

**Figure 1:**
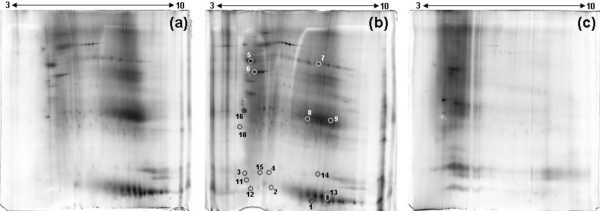
**Representative 2-DE gel map of FFPE adenoma parathyroid tissue proteins**. After deparaffinization in xylene and rehydration through a series of graded alcohols, tissue was resuspended in 20 mM TrisHCl at pH 4 **(A)**, 6 **(B) **or 9 **(C) **with 2% SDS and 0.2 M Glycine. The homogenate was heated at 100°C for 20 min and at 60°C for 2 hours. A total of 150 μg of proteins was separated by 2-DE using 18 cm pH 3-10 NL strip and 12.5% SDS-PAGE. Proteins were detected by silver staining. The map was analyzed by Image master 2D platinum software. The gels were performed in triplicate. The reported gel images are representative of a triplicate for each pH buffer.

From the numerous protocols [7-11] that obtain a high protein recovery, we chose SDS detergent and the heating of samples to favor the hydrolysis of methylene bridges, and the addition of 200 mM glycine to serve as a formaldehyde scavenger. It is known [7] that the pH of the buffer affects proteins solubilization, so in order to establish an optimized extraction method for FFPE parathyroid adenoma samples, we tested extractions at buffer pH values of 4, 6 and 9. The different pH values changed the quality of the protein extracted ions depending on the physical properties of the proteins such as the isoelectric point. Pooled samples were processed, and yields of the proteins extracted with each extraction buffer were determined after examining the number of spots detected with 2-DE. The best results for protein amounts extracted using buffer pH values of 4, 6 and 9 were 54.3, 61.4 and 17.2 μg/slice, respectively. Analysis of 2-DE gels detected 158 ± 15, 186 ± 17 and 68 ± 9 (n = 3) (mean ± SD) protein spots within a non-linear pH range from 3 to 10 for extraction buffers having pH values of 4, 6 and 9, respectively. The number of spots was significantly lower than that obtained from the fresh/frozen tissues as previously described by us [1], but was comparable with that reported for other FFPE tissue extracts [11]. The best results were obtained with extraction buffers at pH 6. Typical 2-DE gel images of FFPE parathyroid protein extracts are shown in Figures 1A, B and 1C. The gel replicates for pH values of 4, 6 and 9 showed a percentage of matched spots ranging from 85 to 95%.

### Liquid chromatography electrospray ionization tandem mass spectrometry (LC-ESI-MS/MS) protein identifications in gel-based and in gel-free proteomic approaches

#### Gel-Based approach

Sixteen protein spots extracted from FFPE adenoma parathyroid tissues using buffer with pH 6 and separated by 2DE were cut and analyzed by mass-spectrometry. LC-ESI-MS/MS analysis identified 37 different proteins with a 100% protein identification probability, and among these 11 identified proteins were present in multiple spots. A list of proteins identified based on estimated gel and theoretical MW and pI, number of matched peptides, best ion score, coverage percentage, and identified peptides, is shown in additional file 1. Spot No. 12 was not identified. Only Spots No. 2, No. 11 and No. 15 were identified as unique proteins, while the others led to the detection of a significant number of peptides belonging to up to nine different proteins.

#### Gel-free approach

**Figure 2:**
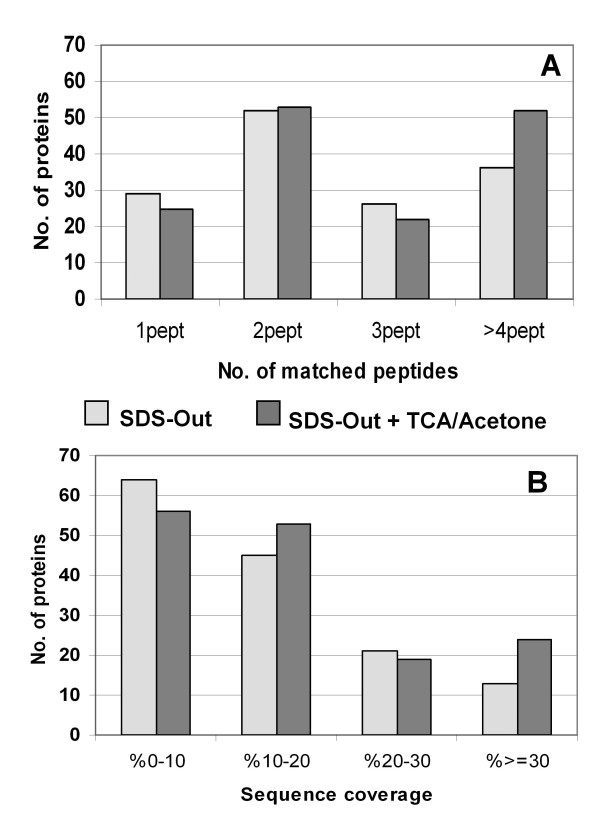
**Gel-free approach: MS/MS sequence coverage and matched peptides**. Histograms representing distribution of number of matched peptides **(A) **and sequence coverage **(B) **for proteins identified in SDS-Out and SDS-Out + TCA/Acetone sample preparations, respectively.

**Figure 3:**
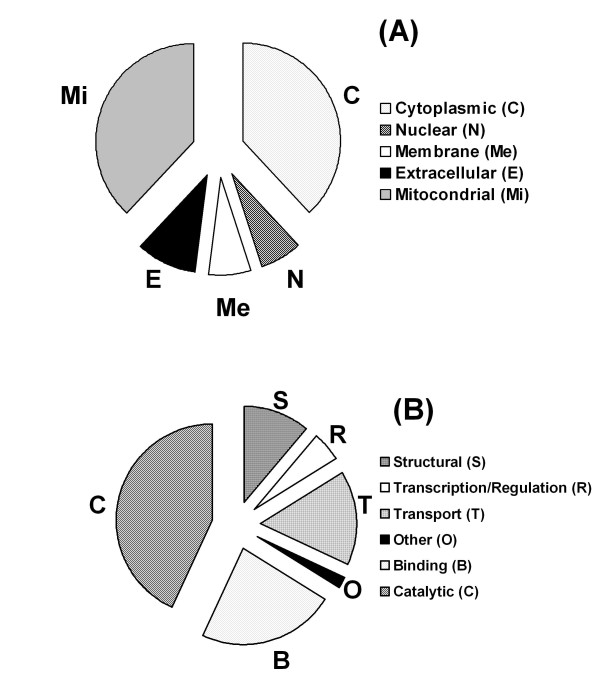
**Subcellular localization and molecular functions**. Subcellular localization and molecular functions of proteins identified in FFPE adenoma tissues extracts by gel-free approaches are represented in panels **A **and **B**, respectively. E, extracellular; C, cytoplasmic; Mi, mitochondrial; N, nuclear; Me, membrane. B, binding; C, catalytic; S, structural; R, transcription/regulation; T, transport; O, other.

The SDS-Out + TCA/Acetone and the SDS-Out protein extracts of FFPE parathyroid adenoma tissues were processed by mass spectrometry. Both samples underwent tryptic digestion and were directly analyzed by LC-ESI-MS/MS. The number of proteins identified with a protein identification probability over 95% were 95 for both SDS-Out + TCA/Acetone (n = 3) and the SDS-Out samples (n = 3) (additional file 2), and 28 and 40 for the separate SDS-Out + TCA/Acetone and SDS-Out samples, respectively (Tables 1 and 2). A list of identified proteins, MW, pI,best ion score, matched peptides and coverage values of the best MS/MS results are shown in Tables 1, 2 and additional file 2. The MW of the identified proteins ranged from 8 (G-protein gamma-12 subunit and ATP synthase subunit e) to 305 kDa (thyroglobulin), while the pI ranged from 4.09 (calmodulin) to 11.36 (histone H4). The best MS/MS results were determined both from the number of peptides found for each identified protein (Figure 2A) and from the distribution of sequence coverage for the identified proteins (Figure 2B). High quality LC-ESI-MS/MS data were obtained, with more than 70 proteins that were identified with at least 2 peptides and with a sequence coverage of about of 10-20%. Figures 3A and 3B show the pie charts of functional classification and the sub-cellular localization of all proteins identified by gel-free methodology. Table 3 shows the 17 proteins that were identified from FFPE tissues and that correspond to the proteins previously found differentially expressed in the adenoma of the fresh/frozen tissues [1].

**Table 1:** Protein spots identified by LC-ESI-MS/MS from FFPE extracts separated by the *SDS-out gel-free *approach with probability over 95%.

Protein name	Accession No	Gene Name	Theoretical		Matched peptides	Coverage (%)
14-3-3 protein ipsilon	P62258	YWHAE	29	4.63	2	9.4
2-oxoglutarate dehydrogenase E1 component, mitochondrial	Q02218	OGDH	116	6.39	2	2.2
3-hydroxyisobutyrate dehydrogenase, mitochondrial	P31937	HIBADH	35	8.38	2	7.1
40S ribosomal protein S5	P46782	RPS5	23	9.73	2	14
40S ribosomal protein S7	P62081	RPS7	22	10.09	2	18
60S acidic ribosomal protein P2	P05387	RPLP2	12	4.42	2	24
ADP/ATP translocase 1	P12235	SLC25A4	33	9.78	2	12
Aspartate aminotransferase, cytoplasmic	P17174	GOT1	46	6.53	2	9.4
ATP synthase subunit b, mitochondrial	P24539	ATP5F1	29	9.37	2	7.8
ATP synthase subunit e, mitochondrial	P56385	ATP5	8	9.34	2	26
Calcium-binding mitochondrial carrier protein Aralar1	O75746	SLC25A12	75	8.57	3	4.9
Creatine kinase B-type	P12277	CKB	43	5.34	3	10
Cytochrome b-c1 complex subunit Rieske-like protein 1	P0C7P4	UQCRFSL1	31	9.04	3	17
Cytochrome c oxidase subunit 4 isoform 1, mitochondrial	P13073	COX4I1	20	9.52	2	16
D-3-phosphoglycerate dehydrogenase	O43175	PHGDH	57	6.29	3	6.2
Decorin	P07585	PE	40	8.75	2	11
Elongation factor 1-alpha 1	P68104	EEF1A1	50	9.10	2	5.2
Elongation factor 2	P13639	EEF2	95	6.41	2	2.9
Enoyl-CoA hydratase, mitochondrial	P30084	ECHS1	31	8.34	2	13
Heterogeneous nuclear ribonucleoprotein H	P31943	HNRNPH1	49	6.74	2	7.6
Isochorismatase domain-containing protein 2, mitochondrial	Q96AB3	ISOC2	22	7.67	4	46
Keratin, type I cytoskeletal 16	P08779	KRT16	51	4.98	5	18
Keratin, type II cytoskeletal 2 epidermal	P35908	KRT2	66	8.07	5	13
Keratin, type II cytoskeletal 6B	P04259	KRT6B	60	8.09	3	11
L-lactate dehydrogenase B chain	P07195	LDHB	37	5.71	3	10
Lumican	P51884	LUM	38	6.16	4	12
Lysosome-associated membrane glycoprotein 1	P11279	LAMP1	45	9.00	2	5.0
Malate dehydrogenase, cytoplasmic	P40925	MDH1	36	6.91	2	8.7
Medium-chain specific acyl-CoA dehydrogenase, mitochondrial	P11310	ACADM	47	8.61	2	8.3
Mitochondrial 2-oxoglutarate/malate carrier protein	Q02978	SLC25A11	34	9.92	5	18
NADH dehydrogenase [ubiquinone] 1 alpha subcomplex subunit 4	O00483	NDUFA4	9	9.42	2	25
Nucleoside diphosphate kinase	Q32Q12	NME1-NME2	33	8.70	2	6.80
Peroxiredoxin-6	P30041	PRDX6	25	6.00	3	17
Proteasome activator complex subunit 1	Q06323	PSME1	29	5.78	2	9.2
Protein kinase C inhibitor-2	Q8WYJ5		14	6.50	3	41
Pyruvate dehydrogenase E1 subunit alpha somatic form mitochondrial	P08559	PDHA1	43	8.35	3	7.9
Ras-related protein Rab-1A	P62820	RAB1A	23	5.93	2	13
Serotransferrin	P02787	TF	77	6.81	2	4.7
Succinate dehydrogenase [ubiquinone] flavoprotein subunit, mitochondrial	P31040	SDHA	73	7.06	2	6.6
Transitional endoplasmic reticulum ATPase	P55072	VCP	89	5.14	3	5.7

**Table 2:** Protein spots identified by LC-ESI-MS/MS from FFPE extracts separated by the *SDS-out + TCA/Acetone *gel-free approach with probability over 95%.

Protein name	Accession No	Gene Name	Theoretical		Matched peptides	Coverage (%)
			MW	pI	SDS-Out +TCA	SDS-Out +TCA
14-3-3 protein theta	P27348	YWHAQ	28	4.68	2	9.0
14-3-3 protein zeta/delta	P63104	YWHAZ	28	4.73	2	11
39S ribosomal protein L39, mitochondrial	Q9NYK5	MRPL39	39	7.56	2	7.7
60S ribosomal protein L7	P18124	RPL7	29	1066	2	8.5
Adenine phosphoribosyltransferase	P07741	APRT	20	5.78	3	20
Adenylate kinase 2, mitochondrial	P54819	AK2	26	7.67	2	11
Aflatoxin B1 aldehyde reductase member 2	O43488	AKR7A2	40	6.70	2	9.5
Cathepsin G	P08311	CTSG	29	11.19	2	11
cDNA FLJ61525, highly similar to Thyroglobulin	B4E153		98	5.50	2	17
Fructose-bisphosphate aldolase A	P04075	ALDOA	39	8.30	4	9.9
Fumarate hydratase, mitochondrial	P07954	FH	55	8.85	2	6.3
Galectin-3-binding protein	Q08380	LGALS3BP	65	5.13	2	4.4
G-protein gamma-12 subunit	Q69YP5	DKFZp762E193	8	9.14	2	38
Guanine nucleotide-binding protein subunit beta-2-like 1	P63244	GNB2L1	35	7.60	2	8.8
Heat shock protein HSP 90-alpha	P07900	HSP90AA1	85	4.94	2	5.1
Ig kappa chain C region	P01834	IGKC	12	5.58	3	48
L-xylulose reductase	Q7Z4W1	DCXR	26	8.33	2	9.4
Macrophage-capping protein	P40121	CAPG	39	5.82	2	10
Mitochondrial import inner membrane translocase subunit Tim8 A	O60220	TIMM8A	11	5.08	2	23
NAD(P) transhydrogenase, mitochondrial	Q13423	NNT	114	8.31	2	2.5
NADH dehydrogenase [ubiquinone] iron-sulfur protein 3, mitochondrial	O75489	NDUFS3	30	6.98	2	9.8
Poly(rC)-binding protein 1	Q15365	PCBP1	37	6.66	2	6.7
Profilin-1	P07737	PFN1	15	8.44	3	23
Puromycin-sensitive aminopeptidase	P55786	NPEPPS	103	5.49	2	2.7
Sideroflexin-1	Q9H9B4	SFXN1	36	9.22	2	17
Transaldolase	P37837	TALDO1	38	6.37	2	7.1
Trifunctional enzyme subunit beta, mitochondrial	P55084	HADHB	51	9.45	2	4.6
Ubiquitin	P62988	RPS27A	9	6.56	2	33

**Table 3:** Proteins identified from FFPE tissues and that correspond to those differentially expressed in the adenoma of the fresh/frozen tissues.

Protein Name	Accession N°	Theoretical Mr/pI	Fold variation adenoma *vs* normal in fresh frozen tissue *	**LC/MS/MS from gel-based (A)**, gel-free (B), or both (C)
Voltage-dependent anion selective channel protein1	P21796	31/8.6	↑ 5.1	B
Cytochrome c oxidase subunit5A, mitochondrial	P20674	17/6.3	↑ 7.8	B
Cytochrome b-cl complex subunit 1	P31930	53/5.9	↓ 2.0	B
Protein S100 - A11	P31949	12/6.6	↑ 2.9	A
Parkinson disease protein 7	B2R4Z1	20/6.3	↑3.4	B
Glyceraldehyde 3-phosphate dehydrogenase	P04406	36/8.6	↑ 2.5	C
Apolipoprotein A-I precursor	P02647	31/5.6	↓ 1.85	B
Ezrin	P15311	69/5.9	↑ 2.3	B
Parvalbumin	P20472	11/4.8	↑ 6.1	A
Serum albumin precursor	P02768	71/5.9	↓ 1.7	B
Serotransferrin precursor	P02787	79/6.8	↑ 2.1	C
Ubiquitin carrier protein (Fragment)	A8MUJ2	16/5.4	↑ 4.8	A
40S ribosomal proteín SA	P08865	33/4.8	↓ 1.7	B
Calmodulin	P62158	17/4.1	↑3.2	B
Annexin A5	P08758	36/4.9	↑ 2.7	B
14-3-3 zeta/delta	P63104	28/4.7	↑ 2.6	C
60 kDa heat shock protein, mitochondrial precursor	P10809	61/5.7	↑ 4.1	B

*See reference 1

### Proteins revealed by western blot

**Figure 4:**
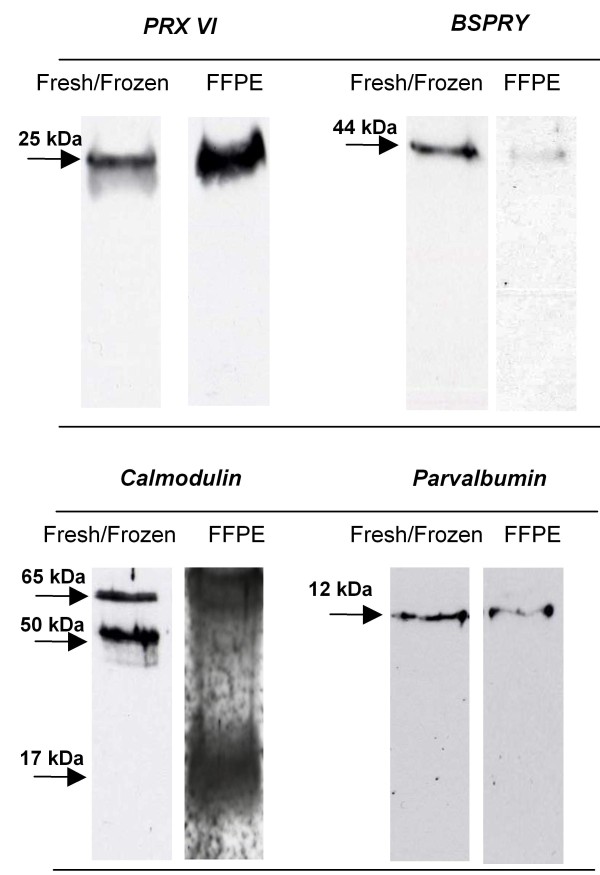
**Western blot analysis**. The presence of PRDX-6, BSPRY, Calmodulin, and Parvalbumin were assessed by immunoblot analysis. Aliquots of protein extracts from FFPE and fresh/frozen adenoma tissues were separated by 1-D SDS-PAGE (12% resolving gel). Proteins were transferred onto nitrocellulose membranes and incubated with specific antibodies. Immunoreactions were evidenced by ECL system.

Western blot analysis with specific antibodies was used to verify the presence of significant proteins previously identified [1] in parathyroid adenoma fresh/frozen tissues. Both FFPE and fresh/frozen tissues samples were analyzed by western blot. The representative immunoblots are shown in Figure 4. Immunoblot results showed that peroxiredoxin 6 (PRDX6), B box and SPRY domain-containing protein (BSPRY), and parvalbumin were present in both the fresh/frozen and the FFPE tissues at the expected molecular weight (MW). In contrast, the anti-calmodulin antibody detected a clear and defined signal at the expected MW (17 kDa) in the FFPE extracts, whereas additional higher reactions (50 and 65 kDa) were detected in the extracts of frozen tissues. No reactions were found for mitogen activated protein kinase 1 (MAPK1), Parkinson disease protein 7 (PARK7), -T-complex protein 1 subunit epsilon (CCT-5) and β-actin.

## Discussion

Recently we have shown the applicability of a proteomic approach to search for potential biomarkers in parathyroid adenomas [1], and our findings represent a promising starting point for the discovery of novel biomarkers in parathyroid carcinoma. However, some problems may occur when studying parathyroid tissues by the proteomic approach, such as the limited number of control specimens and the very low prevalence of parathyroid carcinomas (about 1%). The availability of annotated archival tissue banks is not a resource used for biomarker research, particularly in the validation phase when large sample cohorts are required. The application of proteomic methodologies to FFPE tissues is still at the beginning stages. Many attempts were done to overcome the limits of FFPE sample preparations (*i.e*. protein cross-linking, protein degradation) to obtain a high-throughput extraction methodology which may be reproducible and quantitative. In the present study, protein extracts from parathyroid adenoma FFPE tissues were achieved by treatment at a high temperature, in the presence of a strong detergent and at different buffer pH values. Separation and identification of protein extracts were assessed by gel-based and gel-free proteomic approaches. In the gel-based methodology, the yield in terms of spot numbers resulted very low, and the best results were obtained by using a buffer extraction at pH 6. Based on previously published results [9-11] where 2-DE was used to analyze FFPE tissues, we confirmed the difficulty in obtaining well-resolved protein patterns, in particular for basic proteins. The MS/MS identification of the focused protein spots generated MS spectra of good quality with MS parameters comparable with those derived from proteins of fresh/frozen tissues [1]. Nonetheless, multiple identifications were found for single spots, especially in the high molecular weight and in the acidic regions, depending on an incomplete retrieval of cross-links and protein proteolysis. Therefore, peptides from short residual protein fragments that remain linked could be released due to trypsin digestion and co-identified by MS/MS. Our study shows the applicability of the classical 2-DE methodology. However, the comparison between FFPE extract images and fresh/frozen tissues (data not shown) has suggested that this is not an optimal approach for a proteomic analysis of FFPE tissues. Alternatively, we carried out a gel-free method to analyze protein extracts. Unfortunately, SDS, even in small concentrations impairs the enzymatic digestion of proteins and is not compatible with mass spectrometry analysis. In our study, the use of the SDS-out kit used alone or combined with TCA-acetone precipitation to remove possible detergent residues, has led to comparable results. The efficacy of the SDS-out treatment was confirmed from the absence of real improvements in terms of yield and quality of MS-MS identification after TCA precipitation, suggesting the reliability of this kit in eliminating the anionic detergent. A high number of common proteins were identified in both preparations. The discrepancies observed could be due to several factors such as denaturation, solubility of the proteins, accessibility of tryptic sites, etc., which affected the number of identified peptides. Recently, several studies have been performed using a gel-free approach, but in the majority of cases. a large number of protein identifications have corresponded to a low number of identified peptides [11]. On the contrary, our MS data are encouraging both in the number of identified proteins and in the quality of the MS parameters. Over 70% of proteins were defined by a number of peptides ≥ 2. Functional classification and cellular localization of all 163 identified proteins showed a homogenous distribution of extracted protein classes and of their relative abundance comparable with those previously reported for other tissues [9,10]. Nonetheless, the applicability of a gel-free procedure was supported by the retrieval of 14 of the 30 proteins previously found, differentially expressed in parathyroid adenoma when compared with normal tissues [1]. In this work, we have reported that 22 of the 30 deregulated proteins were clearly over-expressed compared to normal parathyroid. These recent results indicate that 10 of the 22 over-expressed proteins were detected in the gel-free FFPE tissue extracts. This is a very interesting point because it suggests the utility of these extracts as a source in confirming the presence in the parathyroid glands of potential biomarkers of parathyroid diseases. Among the proteins identified, a variety of biological functions were noted including apoptosis, electron transport, response to biotic stimulus, catabolism, lipid metabolism, cell organization, transport, protein metabolism, signal transduction and chaperon activities. Moreover, it is important to point out that proteins not identified by LC/MS/MS, probably due to their low expression, resulted detectable by western blot analysis (*i.e*. BSPRY). However, MAPK1, PARK7 and CCT-5 proteins were not detected in parathyroid adenoma FFPE tissue extracts using their specific antibodies. The lack of immunological detection of these proteins could be related to the alteration of antigenicity in FFPE tissues. Recently, the peculiarities in the antibodies reactivity of FFPE tissues have been investigated by Sompuram *et al*. [31], and in addition to western blot, other antigen-retrieval techniques (protein arrays, ELISA) were tested to detect successfully the protein reactivity in the extracts. In this respect, the choice of antibody is crucial in obtaining the best results.

## Conclusion

Overall, our results are encouraging and suggest the applicability of the proteomic approach to study parathyroid FFPE tissues. In our study, the gel-free procedure gives the best results, and the use of the SDS-out kit is very efficient to remove the anionic detergent and to obtain MS-MS high quality parameters. In addition, these results are comparable with those previously obtained with fresh/frozen parathyroid adenoma samples and demonstrate the potentiality to use these extracts as a source of protein biomarkers both in the research and in the validation phase. With this in mind, it is crucial to achieve reliable results from a quantitative point of view. As far as protein quantification, recent reports have been encouraging [24,32] and indicate the feasibility to assess the differential protein expression in the archived samples and to determine that the magnitudes of differential protein expressions observed in FFPE tissue extracts are comparable to those found in the fresh/frozen samples. We think that the standardization of a method for quantitative proteomic analysis of FFPE tissues is absolutely worth pursuing, and that it could offer new opportunities in identifying-specific biomarkers and their validation using widely available archival samples. This is very important in our case, where the low-prevalence of parathyroid carcinoma is constrained by the availability of fresh/frozen tissues. In the future, we believe that the comparison between parathyroid adenoma and carcinoma FFPE tissue extracts will become realizable.

## Competing interests

The authors declare that they have no competing interests.

## Authors' contributions

AL and FCe conceived of the study; ED and LG carried out the proteomic studies; FC, YDV and GG contributed to the proteomic studies; EP and FS, participated in the diagnosis; FB and LT were involved in the pathology analysis; LG, ED, AL, CM, FCe drafted the manuscript. All authors read and approved the final manuscript.

## Supplementary Material

Additional file 1 **Protein spots identified by LC-ESI-MS/MS from FFPE extracts separated by the gel-based approach**. Click here for file

Additional file 2 **Protein spots identified by LC-ESI-MS/MS from FFPE extracts separated by gel-free approaches with probability over 95%**. Click here for file
