# Supplementary material for: Evaluation of formalin-fixed paraffin-embedded tissues in the proteomic analysis of parathyroid glands
Source: Proteome Sci. 2011 Jun 8;9:29. doi: 10.1186/1477-5956-9-29 (PMC3123619; doi:10.1186/1477-5956-9-29)
Supplement: Additional file 1 — Protein spots identified by LC-ESI-MS/MS from FFPE extracts separated by the gel-based approach. [file 1477-5956-9-29-S1.DOC]

**Protein spots identified by LC-ESI-MS/MS from FFPE extracts separated by the gel-based approach.**

| **Spot No** | **Accession**  **No** | **Protein Name** | **Gene Name** | **Estimated** | | **Theoretical** | | **Matched**  **Peptides** | **Coverage**  **(%)** | **Best ion score** | **Peptides identified** |
| --- | --- | --- | --- | --- | --- | --- | --- | --- | --- | --- | --- |
|  |  |  |  | **MW** | **pI** | **MW** | **pI** |  |  |  |  |
| **1** | **P68871** | Hemoglobin subunit beta | **HBB** | **16** | **6.1** | **16** | **6.74** | **2** | **20** | **59.3** | **(R)FFESFGDLSTPDAVMGNPK(V)** |
|  | **P14174** | Macrophage migration inhibitory factor | **MIF** |  |  | **12** | **7.73** | **2** | **17** | **63.0** | **(M)PMFIVNTNVPR(A)** |
| **2** | **P31949** | Protein S100-A11 | **S100A11** | **16** | **5.2** | **12** | **6.56** | **2** | **26** | **67.4** | **(K)TEFLSFMNTELAAFTK(N)** |
| **3** | **P60660** | Myosin light polypeptide 6 | **MYL6** | **19** | **4.8** | **17** | **4.56** | **5** | **39** | **52.3** | **(K)TEFLSFMNTELAAFTK(N)** |
|  | **P02787** | Serotransferrin | **TF** |  |  | **77** | **6.81** | **7** | **17** | **60.7** | **(K)CDEWSVNSVGKIECVSAETTEDCIAK(I)** |
|  | **O60888** | Protein CutA | **CUTA** |  |  | **19** | **5.42** | **2** | **16** | **32.0** | **(K)TQSSLVPALTDFVR(S)** |
| **4** | **P58876** | Histone H2B type 1-D | **HIST1H2BD** | **19** | **5.2** | **14** | **10.31** | **2** | **19** | **88.9** | **(K)AMGIMNSFVNDIFER(I)** |
|  | **Q5DT20** | Hornerin | **HRNR** |  |  | **282** | **10.04** | **2** | **2.3** | **25.3** | **(R)HGSGSGHSSSYGQHGSGSGWSSSSGR(H)** |
|  | **Q6FI13** | Histone H2A type 2-A | **HIST2H2AA3** |  |  | **14** | **10.9** | **1** | **6.9** | **62.3** | **(R)AGLQFPVGR(V)** |
|  | **P61088** | Ubiquitin-conjugating enzyme E2 N | **UBE2N** |  |  | **17** | **6.13** | **2** | **11** | **24.8** | **(R)ICLDILK(D)** |
|  | **P02766** | Transthyretin | **TTR** |  |  | **16** | **5.52** | **3** | **18** | **45.2** | **(R)KAADDTWEPFASGK(T)** |
| **5** | **P06576** | ATP synthase subunit beta, mitochondrial | **ATP5B** | **56** | **4.9** | **57** | **5.26** | **19** | **56** | **84.0** | **(R)SLDLDSIIAEVK(A)** |
|  | **P07437** | Tubulin beta chain | **TUBB** |  |  | **50** | **4.78** | **8** | **23** | **71.3** | **(K)MAVTFIGNSTAIQELFK(R)** |
|  | **Q5DT20** | Hornerin | **HRNR** |  |  | **282** | **10.04** | **1** | **0.88** | **29.2** | **(R)SGSGWSSSRGPYESGSGHSSGLGHR(E)** |
|  | **P68363** | Tubulin alpha-1B chain | **TUBA1B** |  |  | **50** | **4.94** | **3** | **9.8** | **45.3** | **(R)LDHKFDLMYAK(R)** |
|  | **P01009** | Alpha-1-antitrypsin | **SERPINA1** |  |  | **47** | **5.37** | **5** | **15** | **72.5** | **(K)VFSNGADLSGVTEEAPLK(L)** |
|  | **Q6FI13** | Histone H2A type 2-A | **HIST2H2AA3** |  |  | **14** | **10.90** | **2** | **29** | **56.7** | **(R)VGAGAPVYMAAVLEYLTAEILELAGNAAR(D)** |
|  | **P13861** | cAMP-dependent protein kinase type II-alpha regulatory subunit | **PRKAR2A** |  |  | **46** | **4.96** | **2** | **6.2** | **53.2** | **(R)NISHYEEQLVK(M)** |
|  | **P36957** | Dihydrolipoyllysine-residue succinyltransferase component of 2-oxoglutarate dehydrogenase complex, mitochondrial | **DLST** |  |  | **49** | **9.10** | **2** | **4** | **40.7** | **(R)VLLLDL(-)** |
| **6** | **P06727** | Apolipoprotein A-IV | **APOA4** | **43** | **5.0** | **45** | **5.28** | **3** | **8.8** | **54.9** | **(K)LGEVNTYAGDLQK(K)** |
|  | **P06576** | ATP synthase subunit beta, mitochondrial | **ATP5B** |  |  | **57** | **5.26** | **6** | **18** | **75.2** | **(R)VALTGLTVAEYFR(D)** |
|  | **P62736** | Actin, aortic smooth muscle | **ACTA2** |  |  | **42** | **5.24** | **5** | **29** | **54.2** | **(K)QEYDEAGPSIVHR(K)** |
|  | **Q9BS26** | Thioredoxin domain-containing protein 4 | **ERP44** |  |  | **47** | **5.09** | **2** | **5.2** | **53.5** | **(K)TPADCPVIAIDSFR(H)** |
| **7** | **P62805** | Histone H4 | **HIST1H4A** | **57** | **6.1** | **11** | **11.36** | **1** | **7.8** | **37.2** | **(K)VFLENVIR(D)** |
|  | **P25705** | ATP synthase subunit alpha, mitochondrial | **ATP5A1** |  |  | **60** | **9.16** | **10** | **23** | **69.0** | **(K)TGTAEMSSILEER(I)** |
| **8** | **P68871** | Hemoglobin subunit beta | **HBB** | **28** | **5.9** | **16** | **6.74** | **3** | **30** | **81.4** | **(R)FFESFGDLSTPDAVMGNPK(V)** |
|  | **P58876** | Histone H2B type 1-D | **HIST1H2BD** |  |  | **14** | **10.31** | **1** | **12** | **63.4** | **(K)AMGIMNSFVNDIFER(I)** |
|  | **P62805** | Histone H4 | **HIST1H4A** |  |  | **11** | **11.36** | **2** | **17** | **45.9** | **(R)ISGLIYEETR(G)** |
|  | **Q9BSH5** | Haloacid dehalogenase-like hydrolase domain-containing protein 3 | **HDHD3** |  |  | **28** | **5.22** | **3** | **13** | **57.1** | **(R)LEGILGGLGLR(E)** |
|  | **P60174** | Triosephosphate isomerase | **TPI1** |  |  | **27** | **6.45** | **4** | **18** | **55.2** | **(K)QSLGELIGTLNAAK(V)** |
|  | **P04792** | Heat shock protein beta-1 | **HSPB1** |  |  | **23** | **5.98** | **5** | **40** | **74.4** | **(R)LFDQAFGLPR(L)** |
| **9** | **P68871** | Hemoglobin subunit beta | **HBB** | **28** | **6.2** | **16** | **6.74** | **6** | **54** | **99.4** | **(K)VLGAFSDGLAHLDNLK(G)** |
|  | **P62805** | Histone H4 | **HIST1H4A** |  |  | **11** | **11.36** | **2** | **17** | **44.7** | **(R)ISGLIYEETR(G)** |
|  | **Q9BSH5** | Haloacid dehalogenase-like hydrolase domain-containing protein 3 | **HDHD3** |  |  | **28** | **5.22** | **8** | **42** | **64.9** | **(R)RLEGILGGLGLR(E)** |
|  | **P69905** | Hemoglobin subunit alpha | **HBA1** |  |  | **15** | **8.72** | **2** | **17** | **72.6** | **(K)VGAHAGEYGAEALER(M)** |
|  | **P60174** | Triosephosphate isomerase | **TPI1** |  |  | **27** | **6.45** | **5** | **20** | **68.0** | **(K)VPADTEVVCAPPTAYIDFAR(Q)** |
|  | **P04406** | Glyceraldehyde-3-phosphate dehydrogenase | **GAPDH** |  |  | **36** | **8.57** | **3** | **13** | **65.0** | **(R)VPTANVSVVDLTCR(L)** |
| **10** | **P35030** | Trypsin-3 | **PRSS3** | **23** | **4.8** | **33** | **7.46** | **2** | **7.9** | **58.7** | **(K)VLEGNEQFINAAK(I)** |
|  | **Q5DT20** | Hornerin | **HRNR** |  |  | **282** | **10.04** | **0** | **0** |  |  |
|  | **Q07507** | Dermatopontin | **DPT** |  |  | **24** | **4.70** | **5** | **34** | **102.8** | **(R)AGMEWYQTCSNNGLVAGFQSR(Y)** |
| **11** | **P20472** | Parvalbumin alpha | **PVALB** | **18** | **4.8** | **12** | **4.98** | **2** | **20** | **67.0** | **(K)SGFIEEDELGFILK(G)** |
| **12** |  | N.I. |  | **16** | **4.9** |  |  |  |  |  |  |
| **13** | **P68871** | Hemoglobin subunit beta | **HBB** | **17** | **6.3** | **16** | **6.74** | **7** | **61** | **88.7** | **(R)FFESFGDLSTPDAVMGNPK(V)** |
|  | **P62805** | Histone H4 | **HIST1H4A** |  |  | **11** | **11.36** | **2** | **19** | **35.8** | **(K)VFLENVIR(D)** |
|  | **P69905** | Hemoglobin subunit alpha | **HBA1** |  |  | **15** | **8.72** | **3** | **25** | **65.7** | **(K)VGAHAGEYGAEALER(M)** |
| **14** | **P58876** | Histone H2B type 1-D | **HIST1H2BD** | **19** | **6.2** | **14** | **10.31** | **2** | **19** | **85.5** | **(K)AMGIMNSFVNDIFER(I)** |
|  | **P62937** | Peptidyl-prolyl cis-trans isomerase A | **PPIA** |  |  | **18** | **7.68** | **7** | **45** | **65.5** | **(K)HTGPGILSMANAGPNTNGSQFFICTAK(T)** |
|  | **P30044** | Peroxiredoxin-5, mitochondrial | **PRDX5** |  |  | **22** | **8.85** | **5** | **33** | **106.4** | **(K)ETDLLLDDSLVSIFGNR(R)** |
| **15** | **P58876** | Histone H2B type 1-D | **HIST1H2BD** | **19** | **5.1** | **14** | **10.31** | **2** | **19** | **75.7** | **(K)AMGIMNSFVNDIFER(I)** |
| **16** | **P35030** | Trypsin-3 | **PRSS3** | **27** | **4.8** | **33** | **7.46** | **2** | **7.9** | **54.9** | **(K)VLEGNEQFINAAK(I)** |
|  | **Q5D862** | Filaggrin-2 | **FLG2** |  |  | **248** | **8.45** | **2** | **1.4** | **40.4** | **(R)HQEEESETEEDEEDTPGHKSGYR(H)** |
|  | **P63104** | 14-3-3 protein zeta/delta | **YWHAZ** |  |  | **28** | **4.73** | **5** | **25** | **85.0** | **(K)SVTEQGAELSNEER(N)** |
|  | **P27348** | 14-3-3 protein theta | **YWHAQ** |  |  | **28** | **4.68** | **3** | **26** | **87.1** | **(K)AVTEQGAELSNEER(N)** |
|  | **P28066** | Proteasome subunit alpha type-5 | **PSMA5** |  |  | **26** | **4.74** | **2** | **13** | **68.7** | **(R)AIGSASEGAQSSLQEVYHK(S)** |
|  | **P56537** | Eukaryotic translation initiation factor 6 | **EIF6** |  |  | **27** | **4.56** | **2** | **16** | **39.0** | **(K)TSIEDQDELSSLLQVPLVAGTVNR(G)** |
