# Supplementary material for: Evaluation of formalin-fixed paraffin-embedded tissues in the proteomic analysis of parathyroid glands
Source: Proteome Sci. 2011 Jun 8;9:29. doi: 10.1186/1477-5956-9-29 (PMC3123619; doi:10.1186/1477-5956-9-29)
Supplement: Additional file 2 — Protein spots identified by LC-ESI-MS/MS from FFPE extracts separated by gel-free approaches with probability over 95%. [file 1477-5956-9-29-S2.DOC]

Protein spots identified by LC-ESI-MS/MS from FFPE extracts separated by gel-free approaches with probability over 95%.

| **Protein name** | **Accession No** | **Gene Name** | **Theoretical** | | **Matched peptides** | | **Coverage**  **(%)** | |
| --- | --- | --- | --- | --- | --- | --- | --- | --- |
|  |  |  | **MW** | **pI** | **SDS-Out +TCA** | **SDS-Out** | **SDS-Out +TCA** | **SDS-Out** |
| 10 kDa heat shock protein, mitochondrial | **P61604** | HSPE1 | 11 | 8.89 | 2 | 2 | 24 | 25 |
| 2,4-dienoyl-CoA reductase, mitochondrial | **Q16698** | DECR1 | 36 | 9.35 | 3 | 3 | 14 | 11 |
| 3-hydroxyacyl-CoA dehydrogenase type-2 | **Q99714** | HSD17B10 | 27 | 7.65 | 2 | 4 | 14 | 32 |
| 40S ribosomal protein SA | **P08865** | RPSA | 33 | 4.79 | 2 | 2 | 11 | 11 |
| 60 kDa heat shock protein, mitochondrial | **P10809** | HSPD1 | 61 | 5.70 | 5 | 7 | 14 | 18 |
| 60S acidic ribosomal protein P0 | **P05388** | RPLP0 | 34 | 5.72 | 3 | 1 | 14 | 3.8 |
| Acetyl-CoA acetyltransferase, mitochondrial | **P24752** | ACAT1 | 45 | 8.98 | 3 | 4 | 11 | 14 |
| Aconitate hydratase, mitochondrial | **Q99798** | ACO2 | 85 | 7.36 | 9 | 10 | 16 | 19 |
| Actin, cytoplasmic 1 | **P60709** | ACTB | 42 | 5.29 | 4 | 6 | 15 | 17 |
| ADP/ATP translocase 2 | **P05141** | LC25A5 | 33 | 9.76 | 3 | 4 | 11 | 15 |
| Alpha-1-antitrypsin | **P01009** | SERPINA1 | 47 | 5.37 | 4 | 3 | 12 | 8.1 |
| Alpha-enolase | **P06733** | ENO1 | 47 | 7.01 | 2 | 1 | 6.9 | 4.1 |
| Annexin A5 | **P08758** | ANXA5 | 36 | 4.94 | 4 | 5 | 14 | 17 |
| Apolipoprotein A-I | **P02647** | APOA1 | 31 | 5.56 | 3 | 4 | 15 | 18 |
| Aspartate aminotransferase, mitochondrial | **P00505** | GOT2 | 47 | 9.14 | 3 | 6 | 11 | 19 |
| ATP synthase subunit alpha, mitochondrial | **P25705** | ATP5A1 | 60 | 9.16 | 14 | 14 | 28 | 30 |
| ATP synthase subunit beta, mitochondrial | **P06576** | ATP5B | 57 | 5.26 | 13 | 15 | 38 | 46 |
| ATP synthase subunit f, mitochondrial | **P56134** | ATP5J2 | 11 | 9.70 | 2 | 2 | 26 | 26 |
| ATP synthase subunit g, mitochondrial | **O75964** | ATP5L | 11 | 9.65 | 2 | 2 | 23 | 23 |
| ATP synthase subunit O, mitochondrial | **P48047** | ATP5O | 23 | 9.97 | 4 | 5 | 26 | 30 |
| ATP synthase-coupling factor 6, mitochondrial | **P18859** | ATP5J | 13 | 9.52 | 2 | 3 | 34 | 34 |
| Calmodulin | **P62158** | CALM1 | 17 | 4.09 | 3 | 3 | 31 | 31 |
| Cathepsin D | **P07339** | CTSD | 45 | 6.10 | 3 | 2 | 13 | 6.1 |
| cDNA FLJ60124, highly similar to Mitochondrial dicarboxylate carrier | **B4DLN1** |  | 48 | 9.58 | 1 | 2 | 2.5 | 5.2 |
| Citrate synthase, mitochondrial | **O75390** | CS | 52 | 8.45 | 2 | 2 | 5.8 | 5.8 |
| Cytochrome b-c1 complex subunit 1, mitochondrial | **P31930** | UQCRC1 | 53 | 5.94 | 4 | 4 | 11 | 14 |
| Cytochrome b-c1 complex subunit 2, mitochondrial | **P22695** | UQCRC2 | 48 | 8.74 | 4 | 6 | 10 | 23 |
| Cytochrome c | **P99999** | CYCS | 12 | 9.59 | 2 | 2 | 22 | 18 |
| Cytochrome c oxidase subunit 2 | **P00403** | MT-CO2 | 26 | 4.67 | 2 | 3 | 7.5 | 15 |
| Cytochrome c oxidase subunit 5A, mitochondrial | **P20674** | COX5A | 17 | 6.30 | 3 | 4 | 17 | 41 |
| Cytochrome c oxidase subunit VIb isoform 1 | **P14854** | COX6B1 | 10 | 6.54 | 1 | 4 | 14 | 57 |
| Cytochrome c1, heme protein, mitochondrial | **P08574** | CYC1 | 35 | 9.15 | 1 | 2 | 4.9 | 12 |
| Dihydrolipoyllysine-residue succinyltransferase component of 2-oxoglutarate dehydrogenase complex, mitochondrial | **P36957** | DLST | 49 | 9.10 | 3 | 2 | 11 | 8.4 |
| Electron transfer flavoprotein subunit alpha, mitochondrial | **P13804** | ETFA | 35 | 8.62 | 4 | 3 | 18 | 13 |
| Electron transfer flavoprotein subunit beta | **P38117** | ETFB | 28 | 8.25 | 4 | 7 | 24 | 31 |
| Elongation factor Tu, mitochondrial | **P49411** | TUFM | 50 | 7.26 | 3 | 1 | 7.3 | 3.1 |
| Estradiol 17-beta-dehydrogenase 8 | **Q92506** | HSD17B8 | 27 | 6.09 | 2 | 2 | 11 | 11 |
| Ezrin | **P15311** | EZR | 69 | 5.94 | 3 | 1 | 6.3 | 1.5 |
| Ferritin light chain | **P02792** | FTL | 20 | 5.51 | 3 | 4 | 18 | 29 |
| Fructose-1,6-bisphosphatase 1 | **P09467** | FBP1 | 37 | 6.54 | 3 | 2 | 12 | 9.8 |
| Glucose-6-phosphate isomerase | **P06744** | GPI | 63 | 8.42 | 3 | 2 | 6.8 | 5.6 |
| Glutamate dehydrogenase 1, mitochondrial | **P00367** | GLUD1 | 61 | 7.66 | 6 | 6 | 15 | 15 |
| Glutathione S-transferase P | **P09211** | GSTP1 | 23 | 5.43 | 4 | 2 | 26 | 19 |
| Glyceraldehyde-3-phosphate dehydrogenase | **P04406** | GAPDH | 36 | 8.57 | 5 | 6 | 19 | 25 |
| Haloacid dehalogenase-like hydrolase domain-containing protein 3 | **Q9BSH5** | HDHD3 | 28 | 6.21 | 2 | 4 | 11 | 16 |
| Heat shock 70 kDa protein 1 | **P08107** | HSPA1A | 70 | 5.48 | 5 | 7 | 9.8 | 12 |
| Heat shock cognate 71 kDa protein | **P11142** | HSPA8 | 71 | 5.37 | 5 | 4 | 12 | 11 |
| Heat shock protein beta-1 | **P04792** | HSPB1 | 23 | 5.98 | 3 | 2 | 21 | 13 |
| Heat shock protein HSP 90-beta | **P08238** | HSP90AB1 | 83 | 4.97 | 2 | 2 | 3.2 | 2.9 |
| Hemoglobin subunit alpha | **P69905** | HBA1 | 15 | 8.72 | 2 | 4 | 22 | 51 |
| Hemoglobin subunit beta | **P68871** | HBB | 16 | 6.74 | 6 | 9 | 54 | 80 |
| Heterogeneous nuclear ribonucleoprotein K | **P61978** | HNRNPK | 51 | 5.39 | 2 | 4 | 7.8 | 14 |
| Histone H2A type 1-B/E | **P04908** | HIST1H2AB | 14 | 11.05 | 2 | 6 | 20 | 36 |
| Histone H2B type 1-D | **P58876** | HIST1H2BD | 14 | 10.31 | 4 | 1 | 36 | 12 |
| Histone H3.3 | **P84243** | H3F3A | 15 | 11.27 | 2 | 3 | 13 | 35 |
| Histone H4 | **P62805** | HIST1H4A | 11 | 11.36 | 6 | 7 | 52 | 55 |
| Isocitrate dehydrogenase [NAD] subunit alpha, mitochondrial | **P50213** | IDH3A | 40 | 6.46 | 2 | 2 | 6.3 | 6.0 |
| Isocitrate dehydrogenase [NADP], mitochondrial | **P48735** | IDH2 | 51 | 8.88 | 2 | 2 | 5.8 | 7.3 |
| Keratin, type I cytoskeletal 10 | **P13645** | KRT10 | 60 | 5.13 | 1 | 12 | 1.7 | 28 |
| Keratin, type I cytoskeletal 9 | **P35527** | KRT9 | 62 | 5.14 | 2 | 10 | 4.7 | 35 |
| Keratin, type II cytoskeletal 1 | **P04264** | KRT1 | 66 | 8.15 | 3 | 22 | 8.2 | 40 |
| Malate dehydrogenase, mitochondrial | **P40926** | MDH2 | 36 | 8.92 | 9 | 8 | 38 | 36 |
| Mimecan | **P20774** | OGN | 34 | 5.46 | 2 | 2 | 7.7 | 12 |
| Parathyroid hormone | **P01270** | PTH | 13 | 9.72 | 2 | 1 | 28 | 15 |
| Peptidyl-prolyl cis-trans isomerase A | **P62937** | PPIA | 18 | 7.68 | 3 | 3 | 19 | 16 |
| Peroxiredoxin-2 | **P32119** | PRDX2 | 22 | 5.66 | 4 | 5 | 20 | 27 |
| Peroxiredoxin-5, mitochondrial | **P30044** | PRDX5 | 22 | 8.85 | 2 | 3 | 14 | 18 |
| Phosphatidylethanolamine-binding protein 1 | **P30086** | PEBP1 | 21 | 7.01 | 6 | 7 | 37 | 58 |
| Phosphoglycerate kinase 1 | **P00558** | PGK1 | 45 | 8.30 | 3 | 3 | 10 | 11 |
| Phosphoglycerate mutase 1 | **P18669** | PGAM1 | 29 | 6.67 | 2 | 2 | 14 | 14 |
| Prohibitin | **P35232** | PHB | 30 | 5.57 | 6 | 7 | 28 | 33 |
| Prohibitin-2 | **Q99623** | PHB2 | 33 | 9.83 | 5 | 4 | 19 | 17 |
| Prolargin | **P51888** | PRELP | 44 | 9.47 | 1 | 4 | 2.4 | 13 |
| Protein DJ-1 | **Q99497** | PARK7 | 20 | 6.33 | 2 | 4 | 19 | 37 |
| Pyruvate dehydrogenase E1 component subunit beta, mitochondrial | **P11177** | PDHB | 39 | 6.20 | 2 | 2 | 6.4 | 7.5 |
| Pyruvate kinase isozymes M1/M2 | **P14618** | PKM2 | 58 | 7.96 | 2 | 4 | 5.5 | 10 |
| Sepiapterin reductase | **P35270** | SPR | 28 | 8.25 | 3 | 3 | 17 | 20 |
| Serum albumin | **P02768** | ALB | 69 | 5.92 | 17 | 20 | 31 | 42 |
| Sideroflexin-3 | **Q9BWM7** | SFXN3 | 36 | 9.25 | 3 | 2 | 12 | 8.7 |
| Stress-70 protein, mitochondrial | **P38646** | HSPA9 | 74 | 5.87 | 4 | 6 | 8.5 | 9.4 |
| Succinate-semialdehyde dehydrogenase, mitochondrial | **P51649** | ALDH5A1 | 57 | 8.62 | 2 | 1 | 4.5 | 1.9 |
| Succinyl-CoA ligase [GDP-forming] subunit beta, mitochondrial | **Q96I99** | SUCLG2 | 47 | 6.15 | 3 | 2 | 9.7 | 4.2 |
| Thioredoxin-dependent peroxide reductase, mitochondrial | **P30048** | PRDX3 | 28 | 7.68 | 4 | 3 | 25 | 21 |
| Thyroglobulin | **P01266** | TG | 305 | 5.40 | 40 | 48 | 20 | 24 |
| Transketolase | **P29401** | TKT | 68 | 7.58 | 3 | 2 | 7.9 | 5.9 |
| Trifunctional enzyme subunit alpha, mitochondrial | **P40939** | HADHA | 83 | 9.16 | 4 | 2 | 5.9 | 5.1 |
| Triosephosphate isomerase | **P60174** | TPI1 | 27 | 6.45 | 8 | 7 | 42 | 46 |
| Tripeptidyl-peptidase 1 | **O14773** | TPP1 | 61 | 6.01 | 1 | 2 | 2.5 | 5.7 |
| Tubulin alpha-1B chain | **P68363** | TUBA1B | 50 | 4.94 | 6 | 6 | 21 | 20 |
| Tubulin beta chain | **P07437** | TUBB | 50 | 4.78 | 1 | 4 | 3.4 | 13 |
| Ubiquitin-like modifier-activating enzyme 1 | **P22314** | UBA1 | 118 | 5.49 | 6 | 1 | 9.3 | 1.7 |
| Very long-chain specific acyl-CoA dehydrogenase, mitochondrial | **P49748** | ACADVL | 70 | 8.92 | 1 | 2 | 2.3 | 4.7 |
| Vimentin | **P08670** | VIM | 54 | 5.06 | 2 | 2 | 5.2 | 5.2 |
| Voltage-dependent anion-selective channel protein 1 | **P21796** | VDAC1 | 31 | 8.62 | 5 | 5 | 22 | 25 |
| Voltage-dependent anion-selective channel protein 2 | **P45880** | VDAC2 | 32 | 7.50 | 4 | 5 | 19 | 21 |
|  |  |  |  |  |  |  |  |  |
